# Comparative Anatomy

**Published:** 1877-05

**Authors:** 


### EDITORIAL, ETC.

*Comparative Anatomy.*—Professor Harrison Allen, of the University of Pennsylvania, in a lecture recently delivered, a condensed form of which, prepared by Dr. C. C. Vanderbeck, is published in the *Medical and Surgical Reporter*, speaks of the primary principle and that of evolution as follows:

"ILLUSTRATIONS OF THE PRIMARY PRINCIPLE.—Development depends upon division of functional labor, *i. e.*, the more organs introduced to divide the labor of the body, the higher the animal. This is the best basis for physiological classification. In the very lowest animal only one kind of tissue exists, and the being is almost structureless. Yet all the primary functions, digestion, circulation, respiration, reproduction, excretion, are performed by such an animal. There is no localization or specialization of function; every part of the tissue is capable of performing all the mentioned functions. The amœba, a kind of rhizopod, which in turn belongs to the protozoa division of animals, is a good example. Higher up the scale animals begin to form a skeleton, one of the first differentiation that occurs. An œsophagus appears for the first time in the infusoria, the highest of protozoa. In the low forms of radiates there is no anus proper, the one opening into the cup-shaped alimentary canal answering as both a mouth and an anus. In the higher radiates an independent opening exists for the anus; alimentation and respiration both are performed by one canal in certain molluscs, respiration being carried on in that part equivalent to the pharynx in higher animals. In the human fœtus the intestinal canal exists before the respiratory organs; these may be considered, then, branches of the alimentary canal. Circulation, respiration, and excretion are performed by one organ in some low animals, especially in the entozoa. Thus it is seen that a whole group of functions may be carried on by one system or structure, and that the difference between a high and a low animal is in the division of labor, the differentiation of structure. The liver of the human adult does two things—forms bile, and also sugar, and in the fœtus is also concerned in respiration. Therefore, if we could conceive of a being higher than man, we would probably have the functions of the liver separated. The wolfian bodies in the human fœtus represent, for the most part, both the internal and external organs of generation of the adult. This is only one of the many examples of division of functional labor in the developed body as compared with the fœtus. Another illustration of this first law may be thus condensed: A well-formed kidney does not appear below molluscs; it is absent in articulates below myriapoda. In the ensozoa it it is replaced by the water vascular system.

Principle of Evolution.—Types.—Type, as defined by Professor Agassiz, Dr. Allen thinks is entirely metaphysical. Agassiz taught, it will be remembered, that each type was a divine thought, that each was due to an independent, separate, creative act. Professor Allen is inclined to believe that type is simply a term of convenience; that in reality there is no such thing as type; at any rate, not in the sense used by Agassiz and his followers. It is a convenient word, however, to use in speaking of certain more or less similar forms running through a series of animals. As he has frequent occasion to use the terms general and special type, it will be well to obtain a clear idea of the meaning of these words at the outset. A generalized type is a low form of life; there are few or no attempts at division of functional labor; the specialized type belongs to a higher form of life; specialization or division of function has been carried to a more or less degree of perfection. The first principle is seen, thus, to be the basis of determining whether any given animal is generalized or specialized. The special types are evolved from the general forms. As an illustration of this subject, fish may be mentioned as the most generalized vertebrate. This is told first by the skeleton. The degree of ossification differs throughout the vertebrated series, and is more complete the higher the scale is ascended. In every fish there is a considerable amount of cartilage, all through life. In the amphyoxus the spinal column never becomes bony, but remains cartilaginous all lifetime.

The wolfian bodies probably remain in mature fish; a feature of fetal life of most vertebrates. Again, fish are aquatic, and the lowest class of animals of any grand division lives in this medium. Therefore all aquatic animals are more generalized than terrestrial animals. From a careful study of the structure and life, history, we can quite easily determine, as a rule, the character of the type—whether high or low—of any class of animals. Specialization may be studied in a more restricted sense, as occuring within a given class. Fish, though the most generalized of all vertebrates, have some species that are specialized, addition or change of position of organs to suit particular habits of life. The flounder is flat, and has both eyes on the upper surface. The pike is a highly specialized fish. The coral fish has specialized teeth. The cod-fish is a more generalized form and the amphyoxus is not only the lowest vertebrate, but the most generalized fish. This animal is double-pointed, lives in the sand, and was once thought to be a worm. No bone is found in it. The whole body is of the simplest character. No convolutions exist in the intestines, and there is no sacking for a stomach. All the functions of alimentation are done by this one tissue. The quadrupedal form of mamal is the generalized form. Man, when considered as a whole, is the most specialized mammal, but some particular parts or tissues are more general than the same structures in other mammals. The only mammal that flies is the bat, which is highly specialized for this function.

The teeth of the rodent are highly specialized, especially the incisors; they have no canine, therefore cannot tear. A specialized animal can do only a few things, but can do these few well. The human hand is generally spoken of as the most specialized structure of all animal creation, and the consideration of the hand, its mechanisms and vital endowments, as evincing design, formed one of the series of the Bridgewater treatises. The hand, however, is specialized in its myology, not in its form. The embryonic, therefore the generalized, number of digits, is five. This primal form is retained by man and the elephant; but the hog has only four, two of which are rudimental; the rhinoceros, three; the boa, two; and the horse but one, with concealed rudiments of second and fourth metarsal bones. These animals, consequently, have a more specialized form of a hand, and a foot. In the opossum (itself five-fingered and toed) the muscles of the paw are generalized, being but one flexor muscle, with a rudiment of a superficial. In man the flexor divides into a superficial and deep, a division of functional labor occurs, the muscular system of the hand becomes specialized. Finally, the clavicle is not present in all animals, and its presence indicates a power to bring the tips of the extremities together, and is also indicative of a specialization. This bone is absent in ruminants, imperfect in carnivora, and some rodentia, but perfect in squirrels, etc.

*Individuality* bears a close relationship to the degree of development. If formulated, the law might be expressed, the higher the animal the more marked the individuality. In the same species, one fish cannot be told from another. The same remark is true of corals, and compound animals, such as tape-worm. This latter animal has no mouth, no tail; it is a colony of animals; each segment is an organism, and one mature segment cannot, physiologically, be distinguished from another. The individuality of higher animals is well known; it is seen among sheep, cattle, horses, but in greatest perfection among men. Again, wherever lack of individuality is seen, the risk of death is greatest, and consequently the number of ova and young produced are incomparably in excess of those possessing individuality. Glancing at the downward scale, we are powerful, resist death to a marked degree, but are comparatively few in number. Monkeys are fewer in number than other quadrupeds. Birds are few compared with fish, and so on down the scale. This is a wise law of nature, for those who resist death are not in so much danger of extinction, while those who die easily, or who are the prey of other animals, or whose eggs are destroyed in immense numbers, must produce innumerable eggs and young, in order to protect the species from utter extinction."

